# Salamanders and fish can regenerate lost structures - why can't we?

**DOI:** 10.1186/1741-7007-10-15

**Published:** 2012-02-27

**Authors:** Hans-Georg Simon

**Affiliations:** 1 Department of Pediatrics, Northwestern University, The Feinberg School of Medicine, Children's Memorial Research Center, 2300 Children's Plaza, Chicago, IL 60614, USA

## Abstract

The recent introduction of *in vivo *lineage-tracing techniques using fluorescently labeled cells challenged the long-standing view that complete dedifferentiation is a major force driving vertebrate tissue regeneration. The report in *BMC Developmental Biology *by Juan Carlos Izpisúa Belmonte and colleagues adds a new twist to a rapidly evolving view of the origin of blastemal cells. As classic and recent experimental findings are considered together, a new perspective on vertebrate muscle regeneration is emerging.

See research article http://www.biomedcentral.com/1471-213X/12/9

## Commentary

Loss or serious damage to tissues cannot be repaired - at least not in humans. A severed limb does not grow back, an infarcted heart muscle does not heal by itself. Many animal species do, however, have surprising regenerative abilities. Studies of these natural regenerating systems promise to provide a conceptual understanding of the biology of tissue regeneration, and even partial achievements could revolutionize approaches to regeneration in the clinic.

## How is the vast range of cells and tissues rebuilt during vertebrate regeneration?

Like other organs, vertebrate appendages are composed of complex tissues that originate from multiple germ layers. The limb, for example, consists of epidermis and a peripheral nervous system, both derived from ectoderm, and other internal tissues such as muscle, bone, dermis, and blood vessels, which have a mesodermal origin. In a regeneration-competent vertebrate, damage or complete loss of an appendage initiates a regenerative response that typically involves the early formation of a growth zone of undifferentiated cells, the blastema, at the distal end of the stump. The origin of the newly formed blastemal cells and their fate during the regeneration process have been on-going topics of debate over the past century. Early studies using the regenerating salamander limb and tail indicated that injured multinucleated myofibers can dedifferentiate, give rise to mononucleate progeny, and contribute to the regenerating blastema. Tracing individually labeled myotubes after transplantation documented their capacity to redifferentiate into different lineages within the regenerate, indicating the multipotent nature of derived progenitors cells [1]. Recent advances in generating green fluorescent protein (GFP)-expressing transgenic frogs, salamanders, and fish, combined with molecular marker analyses, have enabled *in vivo *tracking of cells with high precision. Revisiting the open questions concerning the overall contribution and transdifferentiation of lineages, Kragl *et al*. [2] demonstrated that the salamander limb blastema primarily contains lineage-restricted progenitors that remain within their original lineage as they rebuild the lost tissue.

The first demonstration in a vertebrate that different tissues, such as muscle and nerve, are regenerated from distinct progenitor pools came from work on *Xenopus *tadpole tail regeneration [3]. These studies indicated that the activation of muscle-specific stem cells (that is, Pax7+ satellite cells localized adjacent to mature fibers, rather than dedifferentiation, drive muscle regeneration in premetamorphic frogs. In addition, the new study by Rodrigues and colleagues [4] with amputated zebrafish larvae tails produced no evidence of dedifferentiation of the myofibers. Ultrastructural and gene expression data, however, revealed signs of incomplete dedifferentiation in regenerating tadpole tail muscle fibers. This unexpected phenotype might indicate that partial cellular dedifferentiation is sufficient to condition the muscle into a regeneration program, which might not just comprise the myofiber but also could include the activation of satellite cells. A lineage restriction for bone has also been documented in regenerating zebrafish fins, although a cycle of osteoblast dedifferentiation and redifferentiation was demonstrated during blastema formation [5]. In mammals, appendage regeneration is limited to the digit tip, permitting the study of cartilage, bone, epidermal, and nervous tissues but not of muscle tissue as this lineage is not present in this distally amputated tissue. Using the adult limb [6] or neonatal limb model [7], in combination with tissue-specific and inducible mouse cre-reporter lines, these two conceptually similar lineage analyses reached the same conclusion: during mammalian digit-tip regeneration, tissue-resident stem or progenitor cells are fate restricted.

Thus, the recent data from frog, salamander, fish, and mouse models support the hypothesis that lineage restriction during regeneration is the norm. Apparently, each tissue provides a distinct progenitor cell pool to the regeneration blastema, indicating that the vertebrate blastema is a heterogeneous population of cells that have different tissue origins and restricted potentials, which together coordinately regenerate the complex appendage. These studies did not, however, address or conclusively answer the question of whether dedifferentiation occurs within a specific lineage. By contrast, in salamanders, abundant data exist for skeletal muscle dedifferentiation. This finding is supported by recent studies in salamander and zebrafish cardiac muscle regeneration, where dedifferentiation of heart muscle cells results in expansion and redifferentiation to the original cell type [8,9]. Cre/loxP-based lineage tracing to compare the fates of skeletal muscle fibers and satellite cells will be crucial in finally determining the significance of skeletal muscle dedifferentiation versus stem cell activation in this lineage.

## Does muscle have an independent role in controlling the differentiated status?

It is possible that both stem cell activation and dedifferentiation contribute to the production of proliferating progenitors for regeneration. For any specific cell type that acts as a source for new blastemal cells, whether it functions as a stem cell or through dedifferentiation to a progenitor state, a specific molecular programming or reprogramming mechanism must be in place to orchestrate the cellular behaviors that drive the regeneration process. Some evidence that muscle might indeed have a particular position in regeneration has come from investigations on the plasticity of the muscle cell differentiation status. The first experimental evidence that a transcription factor can induce a dedifferentiation response in a mammalian myotube that was thought to be terminally differentiated was reported, a decade ago already, by Odelberg *et al*. [10]. Their key finding was that forced expression of the homeobox protein Msx1 in mammalian myotubes resulted in the fragmentation and generation of mononucleated myoblasts. These findings were then extended by the same group, who demonstrated that the intracellular signaling pathways for dedifferentiation are intact in mammalian cells. Recently, Lehoczky and colleagues [7] proposed a new role for Msx1 as a mediator of bone morphogenic protein (BMP) activity in mouse digit tip regeneration after amputation. Msx1-expressing cells were found to reside in the distal clot, suggesting that the Msx1 protein has a signaling function during regeneration.

The tumor suppressor retinoblastoma protein (RB) has long been known to serve as a cell-cycle gate-keeper, and its natural inactivation by phosphorylation during salamander limb regeneration allows mature muscle cells to dedifferentiate and subsequently enter the cell cycle. While the situation is somewhat more complicated in the mammal, the experimental inactivation of both RB and the alternative reading frame (ARF) tumor suppressor has shown that mammalian muscle cells also can be induced to dedifferentiate and proliferate by the inactivation of these tumor suppressors [11]. These findings in skeletal muscle are echoed by studies in mammalian cardiomyocytes. Engel *et al*. [12] demonstrated that a combination of fibroblast growth factor1 (FGF1) stimulation and p38 mitogen-activated protein (MAP) kinase inhibition can induce dedifferentiation, including contractile apparatus breakdown, following cell proliferation.

Although major inroads have been made over the past years into understanding the mechanisms of cellular reprogramming, especially in creating induced pluripotent stem cells (iPSCs), our knowledge of this process and how it could be applied in the context of regeneration is still in its infancy.

## Which factors should be used to induce terminally differentiated cells to become plastic?

**Figure 1:**
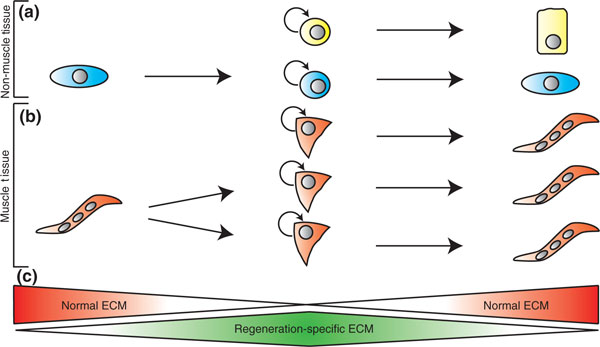
**Origin of blastemal progenitor cells in vertebrate regeneration**. Lineage restriction during vertebrate regeneration has become the accepted norm, but multiple mechanisms might function to generate the blastema cells. **(a) **Non-muscle tissues contain different lineage-restricted (for example, epidermis, nervous system or bone) stem-like cells that produce progenitor cells that have distinct fates. In addition, natural dedifferentiation of a postmitotic cell can generate a proliferation-competent cell within the same lineage, which in turn produces a pool of progenitor cells. **(b) **Skeletal muscle is composed of multinucleated fibers and satellite cells (muscle stem cells). The satellite cells self-renew and produce new differentiated muscle fibers. However, multinucleated muscle fibers can dedifferentiate and fragment to generate a pool of progenitor cells. The dedifferentiation process is accompanied by the disassembly of the sarcomeric contractile apparatus, giving rise to proliferation-competent monocytes, which are similar to satellite cells. These muscle progenitor cells divide and finally differentiate into new muscle. **(c) **Regeneration is accompanied by dramatic reorganization of the tissue extracellular matrix (ECM) environment at the wound site. A regeneration-specific matrix temporarily replaces the normal ECM and differentially directs cellular behaviors, including proliferation, myofiber fragmentation and myoblast fusion. An intriguing hypothesis would be that the regeneration-specific matrix also has a role in balancing dedifferentiation with stem cell activation to produce proliferating progenitors for regeneration.

A close look at the extracellular environment at the wound site might offer some new clues that could help to bring order to the seemingly random array of transcription and signaling factors that appear to control plasticity. Appendage regeneration is characterized by an immediate and dramatic remodeling of all tissues proximal to the site of tissue loss. Recent work from our laboratory revealed a rapid shift from a collagen and laminin-based stiff extracellular matrix (ECM) to a softer transitional matrix that is rich in hyaluronic acid, tenascin-C, and fibronectin [13]. *In vivo *high-resolution three-dimensional imaging revealed this transitional matrix within tissues adjacent to muscle fibers and Pax7+ satellite cells. The use of muscle explants in combination with defined matrix environments further demonstrated that distinct ECM components can differentially direct all of the cellular behaviors necessary for limb regeneration, including proliferation, migration, myofiber fragmentation and myoblast fusion. These findings suggest that the ECM can differentially control cellular behavior during the regeneration process by mediating both growth factor availability and the specific binding of matrices to cell-membrane-localized receptors such as integrins. In this way, the ECM can trigger regeneration-specific gene pathways that are important in the recruitment, expansion, and differentiation of blastema cells (Figure 1).

## Novel approaches to unlock regenerative potential in humans

Recent findings in natural regenerating systems are of great significance because they point to new opportunities to manipulate the local extracellular environment of the wound and possibly to unlock intrinsic regenerative potential by generating new appropriately programmed cells *in vivo*. Following this more natural path either to induce postmitotic cells to dedifferentiate or to activate local stem cell pools would circumvent many of the problems associated with cell transplantation and might lead to the development of new treatments to enhance regenerative wound healing in humans.

## Abbreviations

ECM: extracellular matrix; RB: retinoblastoma protein.

## Acknowledgements

I sincerely thank Dr Shannon J Odelberg for critical reading of and suggestions on the manuscript, and Brandon Holtrup for technical expertise in figure design. Regeneration research in the Simon laboratory was supported through Searle Funds at The Chicago Community Trust and the NIH T90 Regenerative Medicine Training Program.
